# The Animation Lab Brings Molecules to Life

**DOI:** 10.1021/acscentsci.4c00850

**Published:** 2024-06-06

**Authors:** Jonathan Feakins

Janet Iwasa’s interest in animation took shape while she was earning her PhD in cell biology at the University of California, San Francisco. A neighboring lab at UCSF, as it turned out, specialized in motor proteins—including an enzyme called a kinesin, which transports cellular cargo by “walking” along microtubules. In 1999, a member of the lab animated the kinesin’s industrious strut to accompany a paper. After witnessing a finished animation of the spirited locomotion during a joint lab meeting, Iwasa began to wonder what parts of her own research might benefit from a little visual wizardry.

**Figure:**
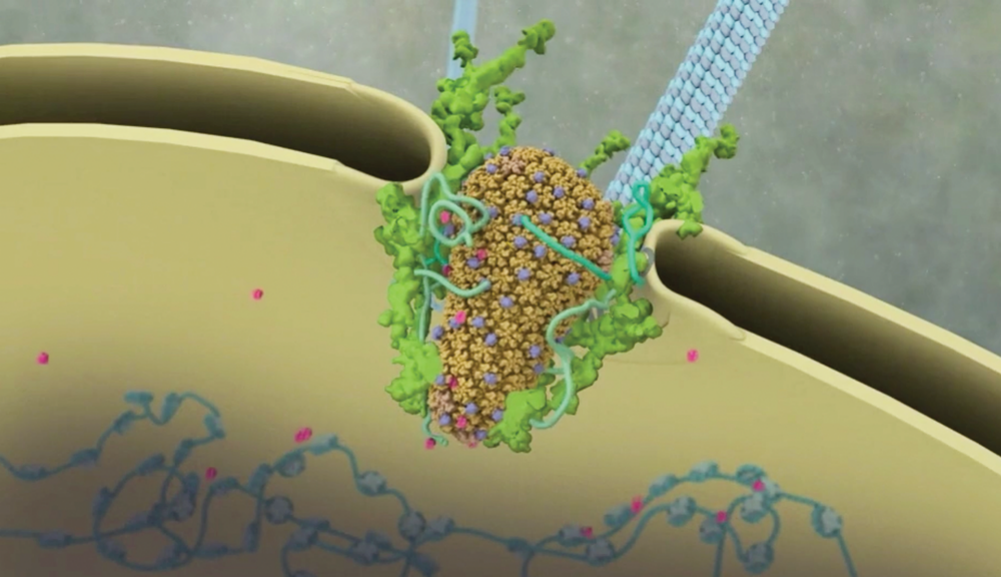
In an animation of the life cycle of HIV, the capsid (HIV’s viral core) is depicted entering the nucleus of a T cell. Credit: Iwasa Lab, University of Utah.

So—in what would become a weekly trek over the course of her PhD work—Iwasa transported herself across town to San Francisco State University (which offered the art classes that her medical university did not). By the time Iwasa finished her PhD in 2006, she’d become so enamored by the art form that she would opine about the light bouncing off a piece of cheese in Pixar Animation Studio’s *Ratatouille*. And when Iwasa joined the ranks of the University of Utah in 2013, she carried her artistic ambitions with her. After spending her first 5 years in research, she finally got the chance to give her art a home.

Founded in 2018, the Animation Lab at the University of Utah may be the only institution of its kind. Here, a squad of dedicated postdoctoral scholars may spend months translating the smallest molecular phenomena—many tinier than the wavelengths of light that would allow them to be “seen”—into a visible, comprehensible spectacle. This unique team enables collaborating researchers to watch their hypotheses escape their mind’s eye and finally take shape in the outside world, live and in color. Iwasa’s lab is one of the first places where prospective molecular animators can not only develop their skills but do so among peers.

“I was working solo, for many years. And I felt pretty isolated,” Iwasa says. “The goal of the lab is to build a community.”

While some other laboratories do focus on visualization, and individual scientists might embrace animation, Iwasa’s lab is—to the best of her knowledge—the only such academic organization that trains postdocs in the skills required to animate scientific discoveries and hypotheses into a form of visual art.

HIV, for instance, bristles with a motley crew of molecular actors, which Iwasa attempted to capture in “The Science of HIV,” a project that has taken her years of work in collaboration with dozens of researchers. In the animation, as the virus approaches a T cell, the cell surface teems with color-coded CD4 and coreceptor proteins reminiscent of a cluster of agitated sea cucumbers. As the virus’s oblong capsid moves toward the T cell’s nucleus, it resembles a malevolent, technicolor eggplant.

While the lab has a more scientific raison d’être than the likes of Pixar or DreamWorks Animation, the field of molecular animation does share a lot of its DNA with its less scientific cousins. Each Animation Lab project, for instance, begins not only with extensive dialogue with the lab’s collaborators but also with an outline and storyboard (which Iwasa prefers to draw by hand).

“If the middle of the storyboard has the most complicated animation, we might start there, and work backward or forward to get the dynamics we want,” says Rachel Torrez, one of the lab’s animators. “It’s not always a linear process.”

**Figure:**
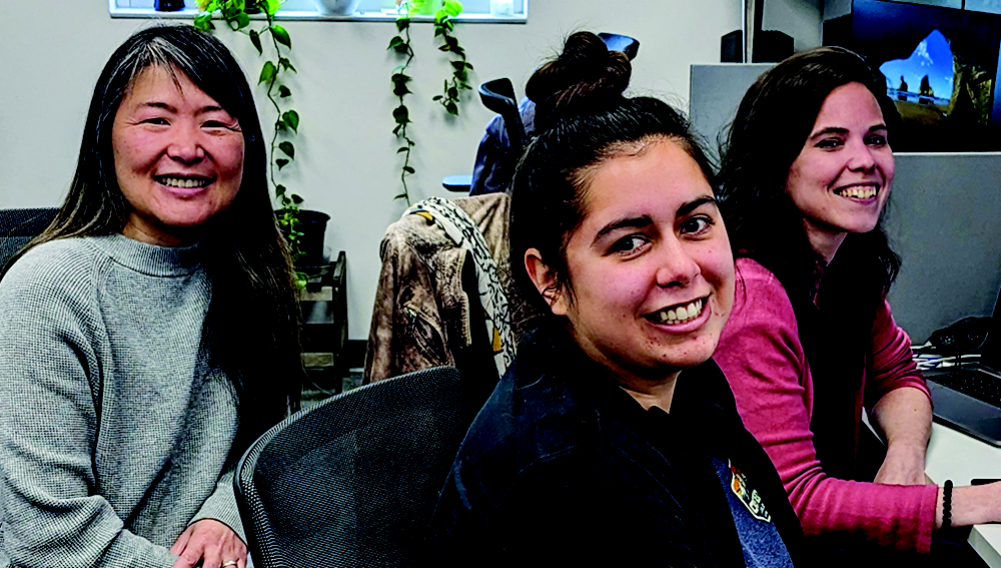
Janet Iwasa (left), Rachel Torrez (center), and Margot Riggi (right) work to animate scientific processes that occur on an infinitesimal scale. Credit: Heidi Schubert.

Torrez, who arrived with a background in cryogenic electron microscopy (cryo-EM), is among the newest members of the Animation Lab team. In a way, the shortcomings of cryo-EM compelled Torrez to attend a molecular animation workshop (that Iwasa happened to be holding).

“Looking at proteins in 3D space, you can learn a lot about their function,” Torrez says. “But near the end of my PhD, you’re just looking at static structures. You have no way of making them move—but you have all this biochemical data that tells you that they’re moving.”

With their specialized skills, the animation team can offer a glimpse into just how mobile these previously static concepts can actually be. But the team’s efforts are often not intended to serve as a definitive, final model. “In many projects, we’re not necessarily trying to build a consensus but rather animate one individual’s view. It’s their vision, their hypothesis, their idea,” says Margot Riggi, a molecular biologist and independent scientific illustrator who has been a member of the Animation Lab since 2020.

But the process of animating these concepts can also serve as a stress test to a researcher’s current hypothesis. Iwasa had one such experience when she worked to animate a type IV pilus structure, which required that she concoct a protein cage above and within the inner membrane of the bacterial cell surface. After laying out the structure, however, Iwasa could not help but notice a sizable roadblock: the proteins would not fit.

“Of course, we can animate anything,” Iwasa explains. “So I had a discussion with [the researchers]: ‘These proteins don’t fit. Do you want me to just make them go right through, or do you want something to happen?’ And they said, ‘Huh.’ ”

The researchers reevaluated their data set and imagined an alternative structure. In the final paper, the authors hypothesized a novel aspect of their model, “in which [the protein] PilC rotates as it assembles the helical pilus fiber.” And while it was not the last time Iwasa’s animations would help shed light on a microscopic world, her creation of the Animation Lab has allowed her—and her team—to shine a spotlight on less-recognized researchers as well.

Since 2020, the Animation Lab has made special efforts to highlight the work of researchers who belong to marginalized racial and ethnic groups. Several times a year, as part of the lab’s #SeeingDiversity series, an animator from the lab pairs up with a scientist of color to produce a visualization of the scientist’s research. Iwasa credits “a very brave student” as the impetus for the project, after the student pointed out that many of Iwasa’s animations to date had primarily featured well-established researchers. “Their question was, ‘How are you helping people who are less established, who are less seen?’ And, you know, that was a hard question to answer,” Iwasa recalls.

Now the Animation Lab has a long list of potential #SeeingDiversity participants, for whom they provide the illustrations free of charge. In some instances, of course, what a participant envisions can stretch the bounds of the team’s skill sets: University of Richmond professor Omar Alberto Quintero-Carmona once asked Iwasa to venture into the word of comic art, to illustrate a myosin in the style of the cover of *Amazing Spider-Man* no. 300, the comic book’s 25th anniversary issue.

**Figure:**
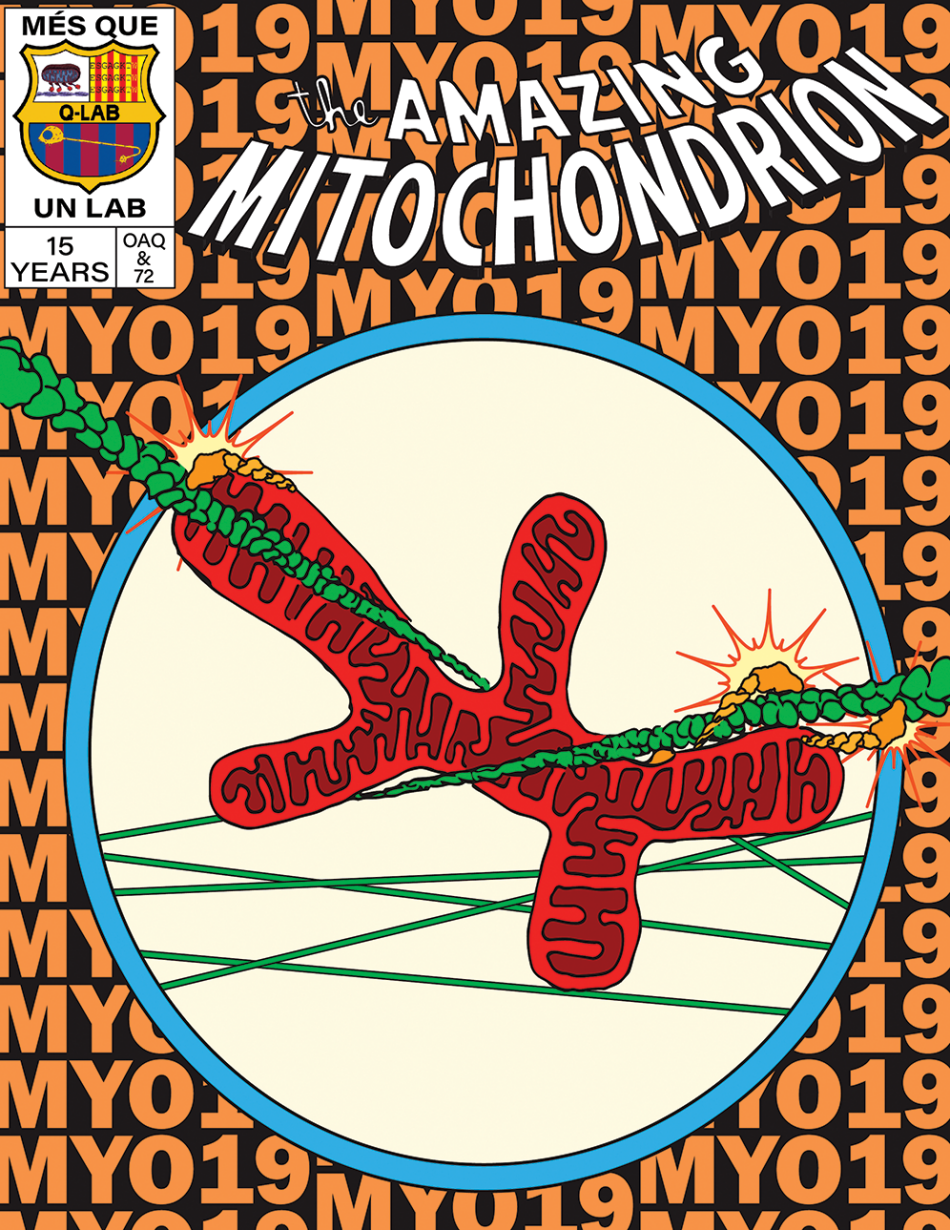
Janet Iwasa and Omar Alberto Quintero-Carmona collaborated to create a comic-style tribute to the humble myosin XIX (MYO19). Credit: Omar Quintero and Janet Iwasa.

Having been in the field almost as long as anyone, Iwasa has a few thoughts on the future of her craft. The lab is currently developing plug-ins that can be added to existing animation software, so that molecular researchers need not experience the same steep learning curve Iwasa (and every member of her team) have had to surmount. “Animation shouldn’t be siloed by specialists, who spend years having to learn software,” Iwasa says. “It should be something that researchers can do.”

Iwasa had actually already released a molecular animation software designed for scientists in 2014, prior to the founding of her lab. But maintaining standalone software requires constant vigilance—the work of a full-time software engineer, essentially—to ensure compatibility with operating systems that are always changing. So while the program (called Molecular Flipbook) did attract users, it could not attract an even more crucial ingredient: dependable funding. A decade later, Iwasa takes care to emphasize that if the broader scientific community hopes to explore animation’s scientific potential, then the groups working in the field will need dedicated financial support.

“People are supportive of visualization—but there are really not many funding opportunities,” Iwasa says. She says this is particularly true if the driving motivation behind such visualization is to foster science communication as outreach; as a result, a majority of the grants the lab secures are part of collaborative efforts with other researchers. While the lab team loves its collaborations, Iwasa believes their work could benefit from dedicated funding that allows the burgeoning field to stand on its own two feet.

“Being able to just explore an idea is a vital part of how research works,” she says, “and should be supported.”

*Jonathan Feakins is a freelance contributor to*Chemical & Engineering News*, the independent news outlet of the American Chemical Society.*

